# Endoscopic injection sclerotherapy versus N-Butyl-2 Cyanoacrylate injection in the management of actively bleeding esophageal varices: a randomized controlled trial

**DOI:** 10.1186/s12876-019-0940-1

**Published:** 2019-02-04

**Authors:** Mohamed A. Elsebaey, Mohamed A. Tawfik, Sherif Ezzat, Amal Selim, Heba Elashry, Sherief Abd-Elsalam

**Affiliations:** 1 Internal Medicine Department, Faculty of Medicine, Tanta University, Tanta, Egypt; 2 Tropical Medicine Department, Faculty of Medicine, Tanta University, Tanta, Egypt

**Keywords:** Esophageal varices, iInjection sclerotherapy, Cyanoacrylate injection

## Abstract

**Background:**

The management of acute esophageal variceal bleeding remains a clinical challenge. Band ligation is the main therapeutic option, but it may be technically difficult to perform in active bleeders. This may necessitate an alternative therapy for this group of patients. This study was conducted to assess the safety and efficacy of sclerotherapy versus cyanoacrylate injection for management of actively bleeding esophageal varices in cirrhotic patients.

**Methods:**

This prospective study included 113 cirrhotic patients with actively bleeding esophageal varices. They were randomly treated by endoscopic sclerotherapy or cyanoacrylate injection as banding was not suitable for those patients due to profuse bleeding making unclear endoscopic visual field. Primary outcome was incidence of active bleeding control and secondary outcomes were incidence of six weeks rebleeding, complications, and mortality among the studied patients.

**Results:**

Initial bleeding control was significantly higher in cyanoacrylate versus sclerotherapy groups (98.25, 83.93% respectively, *P* = 0.007). No significant differences between sclerotherapy and cyanoacrylate groups regarding rebleeding (26.79, 19.30% respectively, *P* = 0.344), complications, hospital stay or mortality rate were observed.

**Conclusions:**

Based on this single-center prospective study, both of these therapies appear to have relatively favorable outcomes, although cyanoacrylate injection may be superior to sclerotherapy for initial control of active bleeding.

**Trial registration:**

[ClinicalTrials.gov Identifier:NCT03388125]–Date of registration: January 2, 2018 “Retrospectively registered”.

## Background

Acute esophageal variceal bleeding is a fatal complication of portal hypertension and one of the main causes of death in cirrhotic patients [1]. The mortality rate of variceal hemorrhage varies from 17 to 57% [2]. HCV-related cirrhosis is the leading underlying etiology of variceal formation and bleeding in Egypt [3, 4].

Endoscopic band ligation is recommended as the main therapeutic modality for bleeding esophageal varices [5], however active variceal bleeding remains a therapeutic challenge to endoscopists as banding may be difficult to be applied in the presence of unclear endoscopic visual field due to massive bleeding [6]. Therefore switching to sclerotherapy, as an alternative therapy to this group of patients, may be used [7].

N-butyl-2-cyanoacrylate is considered the optimal treatment for acute gastric variceal bleeding, but its safety and efficacy in management of bleeding esophageal varices have not been clearly investigated [8]. Moreover, data concerning the best treatment of active esophageal variceal bleeding is limited. To clarify this issue, we performed this study to assess the safety and efficacy of endoscopic injection sclerotherapy versus N-butyl-2-cyanoacrylate injection in the management of actively bleeding esophageal varices in cirrhotic patients.

## Methods

This prospective study was conducted in endoscopy units of Internal Medicine Department, Tanta University Hospital -Nile delta, Egypt - during the period from January 2016 to August 2017.Our hospital represented as the main tertiary referral hospital that received a large number of upper gastrointestinal bleeding (UGIB) cases from many hospitals in the surrounding cities (about 1500–2000 cases/year). One hundred thirteen cirrhotic patients presented with actively bleeding (spurting or oozing blood) esophageal varices were included in this study (they were selected from 520 patients with acute esophageal variceal bleeding). Patients with any of the following: other sources of UGIB than esophageal varices, hepatic encephalopathy or hepatocellular carcinoma (HCC) were excluded from the study.

Patients participating in the study were randomly divided into 2 groups (using consecutively numbered envelopes containing the treatment applied); Sclerotherapy group: included 56 patients who treated by endoscopic sclerotherapy using 5% ethanolamine oleate and Cyanoacrylate group: included 57 patients who treated by endoscopic N-butyl-2-cyanoacrylate injection. The study protocol was done in accordance to the ethical guidelines of the 1975 Helsinki Declaration and was approved by research ethics committee of quality assurance unit in faculty of medicine, Tanta University. A written informed consent was obtained from all included patients in this study.

All patients were subjected to the following: full medical history taking, thorough clinical examination, laboratory investigations including (complete blood count, liver function tests & blood urea and serum creatinine), abdominal ultrasonography and upper gastrointestinal endoscopy. The severity of liver cirrhosis was assessed using Child-Pugh score [9].

### Emergency upper gastrointestinal endoscopy

Before endoscopy, patients were resuscitated. Blood transfusion was done if a hemoglobin level was < 8 g/dl [7]. Platelet transfusion was considered in patients with platelet count< 50 ×  10^9^/L. [10] Ceftriaxone vial (ceftriaxone, Sandoz), IV 1 g/24 h for 5 days and somatostatin (sandostatin, novartis); 100 μg IV as an initial bolus followed by continuous infusion of 25–50 μg/h for 2–5 days, were given to all patients [11, 12].

Upper endoscopy was done once the patient’s hemodynamic permitted. After identification of bleeding source (spurting or oozing esophageal varix), endoscopic haemostatic procedure either by intravariceal injection sclerotherapy (using 5% ethanolamine oleate) or by cyanoacrylate injection was done. In our opinion, inspite of hardly identifying the bleeding source, use of injection sclerotherapy or cyanoacrylate injection has several advantages over band ligation: first, the injection can be performed immediately, i.e. without the need to withdraw the scope, load the banding device, and then reintroduce the scope again. Second, and for the aforementioned reasons, it saves time in this situation where time was precious. Third, the field of view is not narrowed by the attachment of a banding device.

Standardized cyanoacrylate injection technique: N-butyl-2-cyanoacrylate ampoule (0.5 mL) (GluStitch® Twist, GluStitch Inc. Delta, BC, Canada) diluted with 0.8 mL of lipiodol® was injected using Pentax video-endoscopy and a 23-gauge disposable injection needle (Wilson-Cook Medical Inc., USA), immediately followed by 1- 2Ml distilled water to flush out the remaining cyanoacrylate from the dead space of the catheter into the injected varix then the needle was retracted. If the bleeding persisted after the first injection, a second ampoule was injected with the same modality [13]. During the injection procedure, the endoscopist continuously insufflated air with no suction to keep any excess glue away from the tip of the scope. Cyanoacrylate injection required a high degree of experience as its rapid hardening during injection made its application less simple than that of ethanolamine oleate. In addition, if cyanoacrylate extravasates and gets stuck to the endoscope, it will cause permanent damage to its working channel.

Esophageal varices were divided (according to their shape and size) into the following grades: F1: small straight veins, F2: slightly enlarged tortuous veins occupying < 1/3 of the esophageal lumen, F3: large coil- shaped varices that occupied > 1/3 of the esophageal lumen [14].

Follow up: clinical assessment and endoscopic follow up with band ligation were performed every two weeks for six weeks. Primary outcome was incidence of control of active bleeding and secondary outcomes were incidence of rebleeding, complications, and mortality among the studied patients.

Control of active bleeding: was considered when there were no hematemesis, stable hemodynamic status, and stable hemoglobin concentration without blood transfusion for 24-h interval from endoscopy [15].

Rebleeding: was defined as the occurrence of a new episode of hematemesis or melena that associated with hemodynamic instability or a drop in hemoglobin concentration more than 2 g% per day in a previously stable patient [16]. Early rebleeding was considered when bleeding occurred within 5 days of hemorrhage control. Late rebleeding was considered when there was recurrent bleeding between 5 and 42 days [15].

### Statistical analysis

Patients’ data were tabulated and processed using Statistical Program for Social Science (SPSS) version 20.0. Quantitative data were expressed as mean and standard deviation (SD) and were analyzed using unpaired t-test. Qualitative data were expressed as frequency and percent and were analyzed using Chi-square test. In all tests, *p* value was significant when < 0.05. (The full detailed form is: SPSS 20, IBM, Armonk, NY, United States of America). A sample size of 100 patients (50 patients in each treatment group) was calculated to detect a 20% difference in control of bleeding with 80% power at a significance level of *P*-value less than 0.05. The analysis was intention to treat and involved all patients who were assigned randomly.

## Results

This prospective study was conducted in endoscopy units of Internal Medicine Department, Tanta University Hospital - Nile delta, Egypt - during the period from January 2016 to August 2017.

There were no significant differences between sclerotherapy and cyanoacrylate groups as regards the clinical and endoscopic data as shown in Table 1.

**Table 1:** Clinical and endoscopic data of the studied groups

Variables	Sclerotherapy group *N*: 56 (%)	Cyanoacrylate group *N*: 57 (%)	*P*- value
Age (years)			0.101
Range	(30–82)	(28–75)	
Mean ± SD	58.43 ± 9.93	55.26 ± 10.38	
Sex			0.438
Male	42 (75%)	39 (68.42%)	
Female	14 (25%)	18 (31.58%)	
Previous bleeding attacks	28 (50%)	31 (54.39%)	0.641
Child Pugh class			0.878
A	13 (23.21%)	15 (26.32%)	
B	23 (41.07%)	24 (42.11%)	
C	20 (35.71%)	18 (31.68%)	
Hemodynamic instability	47 (83.93%)	42 (73.68%)	0.183
Grade of esophageal varices			0.640
F1	2 (3.57%)	4 (7.02%)	
F2	22 (39.29%)	24 (42.11%)	
F3	32 (57.14%)	29 (50.88%)	

With regard to laboratory investigations, there were no significant differences between the studied groups as shown in Table 2.

**Table 2:** Laboratory investigations of the studied groups

Variables	Sclerotherapy group (N:56) (Range) Mean ± S.D	Cyanoacrylate group (N: 57) (Range) Mean ± S.D	*P*- value
Hemoglobin (g/dl)	(3.6–9.8)	(4.1–10.3)	0.399
	6.87 ± 1.34	7.11 ± 1.62	
WBCs × 10^9^/L	(1.9–8.2)	(2–12.6)	0.142
	4.64 ± 1.54	4.21 ± 1.57	
Platelets × 10^9^/L	(32–172)	(27–214)	0.107
	84.30 ± 33.31	95.61 ± 40.33	
AST (U/L)	(25–169)	(18–147)	0.143
	54.41 ± 26.73	62.96 ± 34.37	
ALT (U/L)	(17–154)	(15–132)	0.120
	46.54 ± 24.36	54.72 ± 30.75	
Serum bilirubin (mg/dl)	(1.2–7.4)	(1.5–8.5)	0.440
	2.34 ± 1.15	2.53 ± 1.46	
Serum albumin (g/dl)	(1.8–3.9)	(1.8–4.1)	0.554
	2.76 ± 0.59	2.69 ± 0.65	
INR	(1.1–2.7)	(1.1–2.8)	0.405
	1.66 ± 0.55	1.58 ± 0.56	
Serum creatinine (mg/dl)	(0.7–3.1)	(0.7–2.8)	0.484
	1.17 ± 0.41	1.22 ± 0.34	
Blood urea (mg/dl)	(19–161)	(16–128)	0.605
	32.29 ± 19.88	34.04 ± 15.76	

*WBCs* white blood cells, *AST* aspartate transaminase, *ALT* alanine transaminase, *INR* international normalized ratio

Control of active bleeding was significantly higher in cyanoacrylate (98.25%) compared to sclerotherapy (83.93%) groups, *P* = 0.007 as shown in Table 3. Among the nine patients whose bleeding did not cease with sclerotherapy; 5 underwent glue injection with immediate control of active bleeding and balloon tamponade was applied in the other 4 patients for 12 h then a second endoscopic therapy using band ligation was done with control of bleeding.

**Table 3:** Post endoscopy outcomes of the studied groups

Variables	Sclerotherapy group *N*: 56 (%)	Cyanoacrylate group *N*: 57 (%)	*P*-value
Control of active bleeding	47 (83.93%)	56 (98.25%)	0.007*
Rebleeding	15 (26.79%)	11 (19.30%)	0.344
Complications:			
Retrosternal pain	12 (21.43%)	6 (10.53%)	0.113
Dysphagia	9 (16.07%)	4 (7.02%)	0.132
Fever	4 (7.14%)	4 (7.02%)	0.979
SBP	3 (5.36%)	2 (3.51%)	0.633
Hospital stay (days)			0.067
Range	(2–17)	(2–13)	
Mean ± SD	4.79 ± 2.85	3.95 ± 1.88	
Mortality	11 (19.64%)	9 (15.79%)	0.592

*means statistical significant, *SBP* spontaneous bacterial peritonitis

The amount of ethanolamine oleate that was used for each patient in sclerotherapy group ranged from 3 to 9 mL, with mean (5.96 ± 1.72). In cyanoacrylate group; the dose of injected cyanoacrylate in each patient ranged from 0.5-1 mL, with mean (0.66 ± 0.235).

**Fig. 1:**
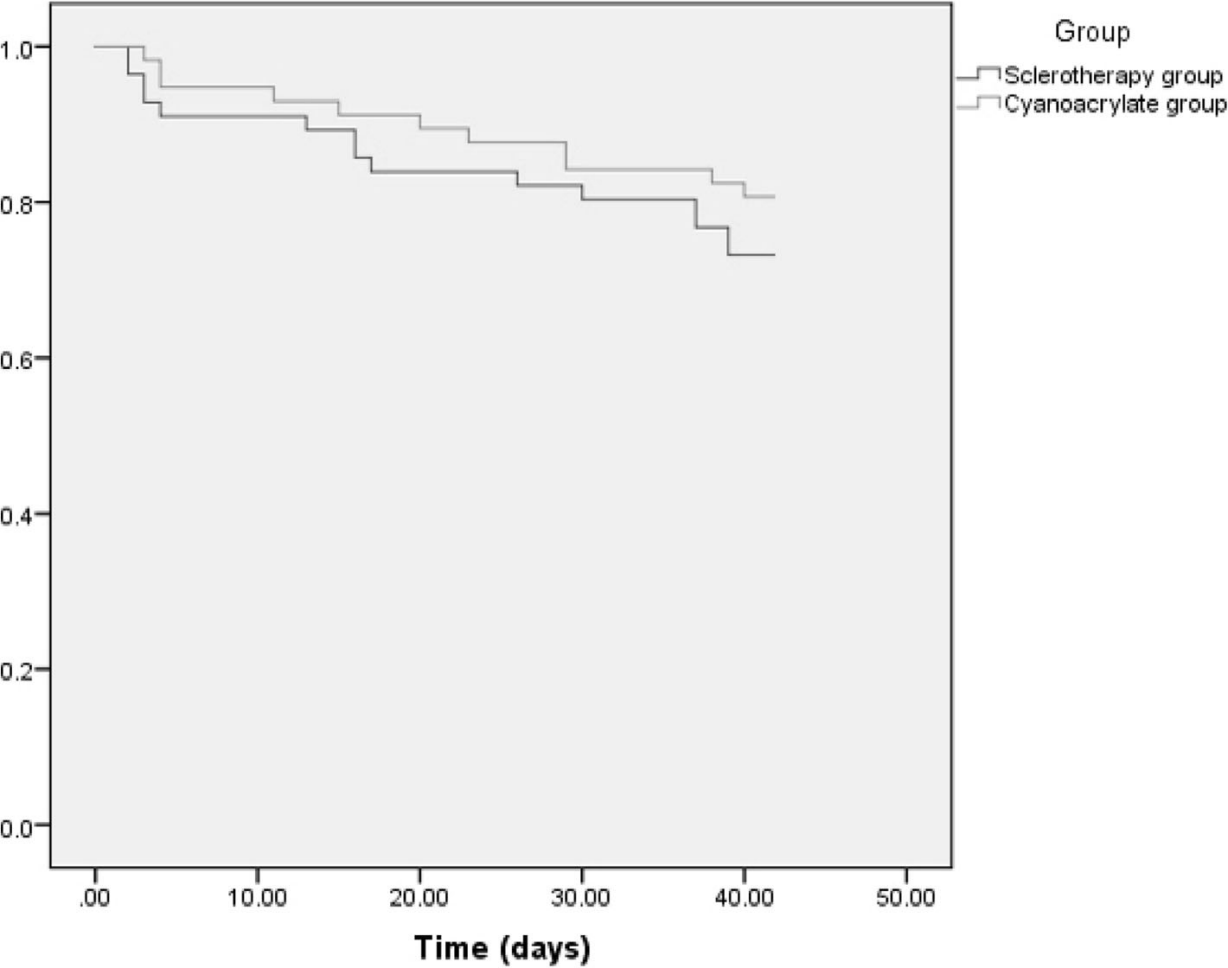
The Kaplan-Meier curve of rebleedings

Rebleeding in sclerotherapy group (26.79%) was higher than that in cyanoacrylate group (19.30%), but without significant difference, *P* = 0.344 as shown in Table 3. Timing, presentation, causes and treatment of rebleeding in the studied groups were shown in Table 4. The Kaplan-Meier curve of rebleeding was shown in Fig. 1.

**Table 4:** Management of rebleeding in the studied groups

Variables		Sclerotherapy group *N*: 15 (%)	Cyanoacrylate group *N*: 11 (%)
Timing	Early rebleeding	5 (33.33%)	3 (27.27%)
	Late rebleeding	10 (66.67%)	8 (72.73%)
Clinical presentation	Hematemesis	8 (53.33%)	6 (54.55%)
	Melena	3 (20%)	3 (27.27%)
	Hematemesis and melena	4 (26.67%)	2 (18.18%)
Causes	Bleeding from the same site and/or post injection esophageal ulcers	7 (46.67%)	2 (18.18)
	Bleeding from other sites (OV&GV)	8 (53.33%)	9 (81.82)
Treatment	Conservative treatment	6 (40%)	3 (27.27%)
	Band ligation	6 (40%)	6 (54.55%)
	Cyanoacrylate injection	3 (20%)	2 (18.18)

*OV* oesophageal varices, *GV* gastric varices

There were no significant differences between the studied groups as regards the incidence of post-endoscopy complications in the form of retrosternal pain, dysphagia, fever or spontaneous bacterial peritonitis (SBP). Regarding hospital stay, there was no significant difference between sclerotherapy (4.79 ± 2.85) and cyanoacrylate (3.95 ± 1.88) groups, *P* = 0.067 as shown in Table 3.

As regards mortality rate, there was no significant difference between sclerotherapy (19.64%) and cyanoacrylate (15.79%) groups, *P* = 0.592 as shown in Table 3. The cause of death in the sclerotherapy group was: uncontrolled bleeding after endoscopy in 2 patients, progressive hepatic failure in 6 patients, SBP in 2 patients and hepatic coma in one patient. While the cause of death in the cyanoacrylate group was: progressive hepatic failure in 6 patients, hepatorenal syndrome in 2 patients and SBP in one patient.

## Discussion

In the present study, 113 out of 520 patients (21.73%) with bleeding esophageal varices had active bleeding at emergency endoscopy. This massive bleeding represented a technical difficulty in identifying and banding the bleeding source, therefore injection sclerotherapy or cyanoacrylate injection was performed as an alternative therapeutic modality. Esmat, et al. [6] reported that 58 out of 151 patients (38.41%) were actively bleeding at endoscopy, profusely enough, to change the decision of treatment from band ligation to sclerotherapy. Cipolletta, et al. [13] stated that 52 out of 133 patients (39.1%) were active bleeders at endoscopy and treated by cyanoacrylate injection.

Regarding control of active bleeding, the current study showed that the initial hemostasis was significantly higher in cyanoacrylate versus sclerotherapy groups (*P* = 0.007).

Initial hemostasis was achieved in 83.93% of our patients who underwent endoscopic sclerotherapy; this was comparable to the results of Esmat, et al. [6] who documented that the rate of initial arrest of variceal bleeding using sclerotherapy was 84.5%. On the other hand, lower percentage (55.6%) of controlling active bleeding was reported by Maluf-Filho, et al. [17].

As reported in this work, initial hemostasis using cyanoacrylate was 98.25%. This high rate of initial hemostasis might be attributed to the nature of cyanoacrylate as it polymerizes and hardens immediately upon contact with blood which makes it ideal for obliterating the varix and immediate control of active bleeding [18]. Similar high rate of controlling active bleeding was documented by LJubičić, et al. [19] and Maluf-Filho, et al. [17] who revealed that immediate hemostasis were 100% of their patients.

In this study, rebleeding rate was higher in sclerotherapy group (26.79%) than that in cyanoacrylate group (19.30%), but without significant difference (*P* = 0.344). The higher rate of rebleeding in sclerothearpy group could be attributed to development of esophageal ulcers in many cases due to large volume of ethanolamine oleate needed to arrest active variceal bleeding. Maluf-Filho, et al. [17] stated that recurrent bleeding within the first six weeks was more frequent in sclerotherapy group (55.6%) comparing with the cyanoacrylate group (11.1%), *P* = 0.01. Maluf-Filho, et al. [17] and Sauerbruch, et al. [20] explained the higher rate of rebleeding following sclerotherapy by development of post sclerotherapy esophageal ulcers in most of their cases. On the other hand, Amer, et al. [21] reported lower percentage of rebleeding rate (15.38%) following injection sclerotherapy but still sclerosant ulcer was the cause of rebleeding in most of their cases.

In the current study, there were no significant differences regarding post endoscopy complications, also there were no complications to staff or endoscope. Only in two cases, cyanoacrylate was stuck to the endoscope lens and the endoscope was immediately withdrawn and the glue was removed by acetone. Blockage to injection needle was experienced in 6 cases during cyanoacrylate injection. Distant embolisation from intravariceal cyanoacrylate injection was not observed in any patient in the present study. This was in accordance with the results of Cipolletta, et al. [13] who documented that cyanoacrylate injection for bleeding esophageal varies was safe to perform with no incidence of distant embolisation. Park, et al. [22] concluded that predisposing factors that increase the risk of embolisation are excessive dilution, large volumes (> 1 mL/injection), and rapid injection of cyanoacrylate.

As regard six weeks mortality rate in the present study, there was no significant difference between sclerotherapy (19.64%) and cyanoacrylate (15.79%) groups, *P* = 0.592. The cause of death in most cases was related to the severity of underlying liver disease as most of deceased patients were Child Pugh class C.

Esmat, et al. [6] reported that six weeks mortality rate of actively bleeders who treated by injection sclerotherapy was 36.2%. Cipolletta, et al. [13] stated that mortality rate was 15.4% following endoscopic therapy by cyanoacrylate injection. On the other hand, high rates of mortality (72.2% versus 33.3% in sclerotherapy and cyanoacrylate groups respectively) were observed by Maluf-Filho, et al. [17] and this was explained by that all their patients were Child-Pugh class C.

In this study, cyanoacrylate injection was superior to sclerotherapy for control of active bleeding despite smaller volume used. Perhaps this was because cyanoacrylate polymerized and hardened within 20 s upon contact with blood owing to the presence of ions and proteins, resulting in immediate control of hemorrhage [16]. Most of hazards of glue can be avoided when it was used by well-experienced hands with adherence to a standardized injection technique as mentioned before in methodology.

There were some limitations to this work; it was carried out in a single center, small sample size and short follow up period. Another limitation was exclusion of the patients with hepatic encephalopathy as they would be at high risk of aspiration during endoscopy that necessitates endotracheal intubation to protect their airways. Meanwhile, in this study we used only conscious sedation during performing endoscopy without need to intubation.

## Conclusions

Based on this single-center prospective study, both of these therapies appear to have relatively favorable outcomes, although cyanoacrylate injection may be superior to sclerotherapy for initial control of active bleeding. We recommended that both injection sclerotherapy and cyanoacrylate injection should be considered in patients with actively bleeding esophageal varices when band ligation is technically difficult.

## Abbreviations

ALT: Alanine transaminase
AST: Aspartate transaminase
GV: Gastric varices
HCC: Hepatocellular carcinoma
HCV: Hepatitis C virus
INR: International normalized ratio
OV: Oesophageal varices
SBP: Spontaneous bacterial peritonitis
SD: Standard deviation
SPSS: Statistical Program for Social Science
UGIB: Upper gastrointestinal bleeding
WBCs: white blood cells
